# High risk for obstructive sleep apnea and other sleep disorders among overweight and obese pregnant women

**DOI:** 10.1186/s12884-015-0633-x

**Published:** 2015-09-02

**Authors:** Jayne R. Rice, Gloria T. Larrabure-Torrealva, Miguel Angel Luque Fernandez, Mirtha Grande, Vicky Motta, Yasmin V. Barrios, Sixto Sanchez, Bizu Gelaye, Michelle A. Williams

**Affiliations:** Department of Epidemiology, Harvard T.H. Chan School of Public Health, 677 Huntington Ave, K501, Boston, MA, 02115, USA; Instituto Nacional Materno Perinatal de Lima, Lima, Peru; Departamentos de Medicina y Ginecología y Obstetricia Universidad Nacional Universidad Nacional Mayor de San Marcos, Lima, Peru; Universidad de Ciencias Aplicadas, Lima, Peru; Asociación Civil de Proyectos en Salud, AC.PROESA, Lima, Peru

## Abstract

**Background:**

Obstructive sleep apnea (OSA), a common and serious disorder in which breathing repeatedly stops during sleep, is associated with excess weight and obesity. Little is known about the co-occurrence of OSA among pregnant women from low and middle-income countries.

**Methods:**

We examined the extent to which maternal pre-pregnancy overweight or obesity status are associated with high risk for OSA, poor sleep quality, and excessive daytime sleepiness in 1032 pregnant women in Lima, Peru. The Berlin questionnaire was used to identify women at high risk for OSA. The Pittsburgh Sleep Quality Index (PSQI) and Epworth Sleepiness Scale (ESS) were used to examine sleep quality and excessive daytime sleepiness, respectively. Multinomial logistic regression procedures were employed to estimate odds ratios (aOR) and 95 % confidence intervals (CI) adjusted for putative confounding factors.

**Results:**

Compared with lean women (<25 kg/m^2^), overweight women (25–29.9 kg/m^2^) had 3.69-fold higher odds of high risk for OSA (95 % CI 1.82–7.50). The corresponding aOR for obese women (≥30 kg/m^2^) was 13.23 (95 % CI: 6.25–28.01). Obese women, as compared with their lean counterparts had a 1.61-fold higher odds of poor sleep quality (95 % CI: 1.00–2.63).

**Conclusion:**

Overweight or obese pregnant women have increased odds of sleep disorders, particularly OSA. OSA screening and risk management may be indicated among pregnant women in low and middle income countries, particularly those undergoing rapid epidemiologic transitions characterized by increased prevalence of excessive adult weight gain.

## Background

Obesity continues to be one of the fastest growing metabolic conditions worldwide. According to the World Health Organization (WHO) the prevalence of obesity is estimated to be approximately 12 % making it one of the 21^st^ century epidemic disease [1]. The prevalence of obesity in Andean Latin American countries has almost doubled from 9 % in 1980 to 17 % in 2008 [2]. Recent studies conducted in Peru found that more than 40 % of the adult population in the metropolitan Lima is overweight or obese [3]. Excess consumption of energy dense foods and lack of physical activity have been implicated as reasons for the rise in obesity prevalence in Peru and elsewhere. Major obesity-related chronic disorders include: cardiovascular diseases, diabetes, hypertension, and breathing difficulties [1]. During pregnancy, maternal overweight or obese status are associated with adverse perinatal and neonatal outcomes such as gestational diabetes [4], pregnancy induced hypertension, preeclampsia [5], cesarean delivery [6], miscarriage [7], large for gestational age [4], macrosomia [8], and stillbirth [9].

There is a growing body of evidence that documents the impact of obesity on sleep disorders, more specifically obstructive sleep apnea (OSA) [2, 10, 11]. OSA is a condition characterized as repeated episodes of complete or total blockage of the upper airway during sleep [12, 13]. Snoring, persistent daytime sleepiness, and periods of awakening out of breath during the night are hallmark symptoms of OSA [13]. Epidemiologic studies have shown that OSA and poor sleep quality are independently associated with weight gain, cardiometabolic disorders, cognitive impairments, hypertension, psychiatric disorders and headaches [2, 14]. Accumulating evidence also documents a strong, positive and bi-directional association of obesity with high risk for OSA and other sleep disorders [2, 15]. Investigators have argued that increased fat deposits around the upper airways of overweight and obese individuals can produce obstruction of breathing, reducing the flow of oxygen leading to sleep apnea [16]. Conversely, OSA can contribute to obesity because fragmented and non-restorative sleep related to sleep apnea has been associated with increased caloric intake [15]. Sleep patterns are known to change throughout pregnancy in part due to physiological and hormonal changes [17]. Studies have reported increased snoring and narrower upper airways during the third trimester of pregnancy compared to postpartum [18].

Despite the high prevalence of sleep disorders among pregnant women [19] and despite a well-established body of evidence showing elevated BMI as a significant predictor of sleep disorders [20], few studies have investigated the risk of sleep disorders among overweight and obese pregnant women. To fill this void in the literature, we sought to assess the extent to which pre-gestational overweight and obesity status are associated with increased odds of high risk of OSA and other sleep disorders among pregnant women in Peru.

## Methods

### Participants, sample size and study setting

This study was conducted among pregnant women attending prenatal care clinics at the Instituto Nacional Materno Perinatal (INMP) in the city of Lima, Peru between February 2013 and March 2014. The INMP, overseen by the Peruvian Ministry of Health, is the primary referral hospital for maternal and perinatal care. Eligible women were 18 years of age or older, could speak and read Spanish, and with a gestational age between 24 to 28 weeks. Enrolled participants were invited to participate in an interview where trained research personnel used a structured questionnaire to elicit information regarding maternal socio-demographic, lifestyle characteristics, medical and reproductive histories, and sleep characteristics. Anthropometric measures and vital signs were measured by experienced midwives. Women were weighed in light clothing using the WHO standard guidelines. All participants provided written informed consent and study procedures were approved by institutional review boards of the INMP, Lima, Peru and the Harvard T.H. Chan School of Public Health Office of Human Research Administration, Boston, MA, USA.

### Instruments and variable specification

The Berlin Questionnaire originated from Berlin, Germany is a widely used and validated screening instrument for assessing high risk for obstructive sleep apnea (OSA). The questionnaire consists of 11 questions separated into three sections [21, 22]. Section 1 asked participants whether they snore. Those who responded affirmatively were asked how loud their snoring was, how often it occurred, and whether their snoring bothered other people. In the present study, participants were also asked whether anyone has ever noticed cessation of their breathing during sleep. Section 2 asked participants how often they felt tired or fatigued right after sleep, how often they felt tired, fatigued, or not up to par during wake time, and whether they ever fall asleep driving a car. In section 3, participants were asked about a history of hypertension, as well as their height, weight, and age. A section was considered positive if there were two affirmative answers in either section 1 or 2, or one affirmative response in section 3. In section 3, high risk for OSA was defined when there was a history of hypertension or obesity.

The Berlin questionnaire is widely used in pregnancy [23–26]. For the purposes of this study when 2 or more sections were classified as positive, the participant was deemed to be at high risk for OSA [21, 22]. In addition, given the lack of consensus concerning the utility of this diagnostic criteria in pregnancy [23–25], we evaluated the extent to which snoring (i.e., those positive for section 1) is associated with maternal obesity status.

Sleep quality was evaluated using the Pittsburgh Sleep Quality Index (PSQI), a 19-item self-reported questionnaire that assesses sleep quality over the past month [27]. The PSQI has seven sleep components: sleep duration, disturbance, latency, habitual sleep efficiency, use of sleep medicine, daytime dysfunction due to sleepiness and overall quality of sleep. Each component produced a score ranging from 0 to 3, where a score of 3 indicates the highest level of dysfunction. A global sleep quality score is obtained by summing the individual component scores (range 0 to 21) with higher scores indicative of poorer sleep quality during the previous month. Participants with global scores that exceed 5 are classified as poor sleepers [27]. Those with a score of 5 or less were classified as good sleepers. This classification system is consistent with prior studies of pregnancy including those conducted in Peru [28–30].

Daytime sleepiness was measured using the Epworth Sleep Scale (ESS) [31]. The instrument has been widely validated globally including in Peru [32]. The ESS is an 8-item questionnaire capturing an individual’s propensity to fall asleep during commonly encountered situations on a scale from 0 to 3. Overall scores range between 0 and 24. ESS scores of 10 or higher are indicative of excessive daytime sleepiness [31].

### Other covariates

Maternal age at the time of interview was categorized as follows: 18–20, 20–29, 30–34, and ≥35 years. Other sociodemographic variables were categorized as follows: maternal and paternal educational attainment (≤6, 7–12, and >12 completed years of schooling); marital status (married and living with partner vs. others); access to basic foods (very hard/hard, somewhat hard, not very hard); food insecurity (yes vs. no); access to medical care (very hard/hard, somewhat hard, not very hard); and parity (nulliparous vs. multiparous). Pre-pregnancy body mass index was calculated as weight (in kilograms) divided by the square of height (in meters and used to identify lean (BMI < 25 kg/m^2^), overweight (BMI: 25–29.9 kg/m^2^), and obese (BMI ≥ 30 kg/m^2^) women according to the World Health Organization (WHO) criteria.

### Statistical analysis

We examined the distributions of maternal sociodemographic, reproductive, and medical characteristics according to pre-pregnancy BMI categories. Multivariate multinomial logistic regression models were fitted to estimate adjusted odds ratios [33] and 95 % confidence intervals (95 % CI) of sleep disorders in relation to maternal overweight and obesity status after adjusting for potential confounders. Separate models were fitted for each sleep complaint. In multivariable models we adjusted for maternal age, educational attainment, marital status, and parity. Additional adjustment for the other covariates listed in Table 1 did not substantially change odds ratios. We explored the possibility of a nonlinear relation between BMI and odds of high risk for OSA, using generalized additive logistic regression modeling procedures (GAM). Finally, on the basis of study findings reporting limited clinical utility of the total score of the Berlin questionnaire in pregnancy [25], we conducted *post-hoc* analyses to examine the extent to which maternal overweight and obesity status are associated with increased odds of snoring (as assessed using information from section 1 of the Berlin questionnaire). All analyses were performed using Stata 12.0 statistical software (Stata, College Station, TX). The GAM analyses were performed using “R” software version 3.1.2. All reported p-values are two-tailed and deemed statistically significant at α = 0.05.

**Table 1:** Maternal socio-demographics characteristics

	Total sample (*N* = 1,032)
Characteristic	n (%)
Maternal Age (years)	
<19	42 (4.1)
20–29	551 (53.4)
30–34	237 (23.0)
≥35	202 (19.5)
Maternal Age (years) Mean (SD) [Min, Max]	28.6 (6.2) [18, 45]
Marital Status	
Married or living with partner	903 (87.6)
Single or living alone/divorced	128 (12.4)
Maternal Education (years)	
>12	32 (3.10)
7–12	541 (52.4)
≤6	459 (44.5)
Paternal Education (years)	
>12	23 (2.2)
7–12	566 (55.4)
≤6	433 (42.4)
Pre-pregnancy BMI (kg/m^2^)	
<25 (Normal weight)	573 (55.5)
25–29.9 (Overweight)	350 (33.9)
≥30 (Obese)	109 (10.6)
Difficulties to Pay for Basics	
Very hard/hard	153 (14.9)
Somewhat hard	351 (34.1)
Not very hard	526 (51.1)
Food Insecurity	
No	369 (35.8)
Yes	663 (64.2)
Difficulties to Access Medical Care	
Very hard/hard	199 (19.3)
Somewhat hard	714 (69.3)
Not very hard	118 (11.4)

## Results

The socio-demographic characteristics of the participants are presented in Table 1. A total of 1032 pregnant women between the ages of 18 and 45 years (mean age = 28.6 years, standard deviation = 6.2 years) participated in the study. The majority of participants were married or living with their partner (87.6 %) while more than half (55.5 %) reported an education attainment of 7 or more years. Approximately 50 % of participants reported having difficulty paying for basics (48.9 %), while 64.2 % reported food insecurities and more than three fourth (88.6 %) reported difficulties to accessing medical care.

**Fig. 1:**
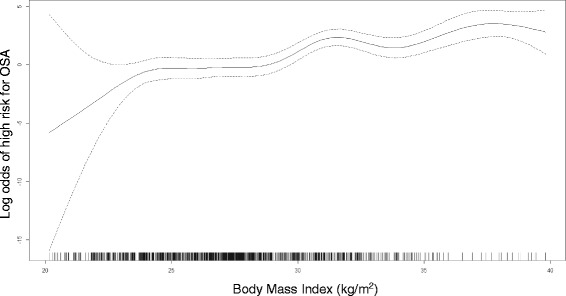
Relation between log odds of high risk for obstructive sleep apnea and pre-pregnancy body mass index (solid line) with 95 % confidence interval (dotted lines)

Table 2 shows the relationship between pre-pregnancy BMI and sleep disorders. The prevalence of high risk for OSA was 2.1 %, 8.0 % and 25.7 % for lean, overweight and obese study participants, respectively. The corresponding prevalence for poor sleep quality were 19.0 %, 21.4 %, and 28.4 %. Generally similar prevalence of excessive daytime sleepiness was noted among lean (12.7 %), overweight (13.1 %) and obese (12.9 %) study participants. In general, as pre-pregnancy BMI increased, the odds of sleep disorders increased. After adjusting for confounders compared with normal weight women (<25 kg/m^2^), overweight women (25–29.9 kg/m^2^) had 3.69-fold higher odds of experiencing high risk for OSA (assessed using the Berlin questionnaire) (95 % CI: 1.82–7.50). Obese women (≥30 kg/m^2^) had a 13.2-fold higher odds of experiencing high risk for OSA (aOR = 13.23; 95 % CI: 6.25–28.01) as compared with their lean counterparts. Additionally the odds of high risk for OSA was modeled in relation to BMI expressed as continuous variable using procedures based on a general additive model. Results from these analyses confirmed a linear relationship between increasing BMI and the odds of high risk for OSA (Fig. 1).

**Table 2:** Odds of obstructive sleep apnea, poor sleep quality, and excessive daytime sleepiness in relation to pre-pregnancy body mass index (BMI)

	Pre-pregnancy BMI in kg/m^2^			Unadjusted OR (95 % CI)		^*^Adjusted OR (95 % CI)	
	BMI <25 (*N* = 573)	BMI 25–29.9 (*N* = 350)	BMI ≥ 30 (*N* = 109)	BMI 25–29.9	BMI ≥30	BMI 25–25.9	BMI ≥30
Berlin Questionnaire	n (%)	n (%)	n (%)				
Low risk OSA	561 (97.9)	322 (92.0)	81 (74.3)	Reference	Reference	Reference	Reference
High risk OSA	12 (2.1)	28 (8.0)	28 (25.7)	4.06 (2.04–8.10)	16.2 (7.90–33.04)	3.69 (1.82–7.50)	13.23 (6.25–28.01)
Poor Sleep Quality							
No (PSQI ≤5)	464 (81.0)	275 (78.6)	78 (71.6)	Reference	Reference	Reference	Reference
Yes (PSQI >5)	109 (19.0)	75 (21.4)	31 (28.4)	1.20 (0.83–1.61)	1.70 (1.06–2.70)	1.16 (0.82–1.63)	1.61 (1.00–2.63)
Excessive Daytime Sleepiness							
No (ESS <10)	500 (87.3)	304 (86.9)	95 (87.1)	Reference	Reference	Reference	Reference
Yes (ESS ≥10)	73 (12.7)	46 (13.1)	14 (12.9)	1.03 (0.70–1.54)	1.00 (0.55–1.86)	1.06 (0.70–1.60)	1.07 (0.57–2.03)

^*^Adjusted for maternal age, education, marital status and parity; Reference group: BMI < 25

*OR* Odds ratio; *CI* Confidence Interval; *OSA* obstructive sleep apnea; *PSQI* Pittsburgh sleep quality index; *ESS* Epworth sleepiness scale

Compared with lean women, overweight women were associated with modest elevated, and statistically non-significant, odds of poor sleep quality (aOR = 1.16; 95 % CI: 0.82–1.63). However, obese women had a statistically significant 1.61-fold increased odds of poor sleep quality (95 % CI: 1.00–2.63), as compared with lean women (Table 2). We observed no clear evidence of an association of pre-pregnancy BMI and excessive daytime sleepiness. In *post-hoc* analyses restricted to the snoring components of the Berlin questionnaire (section 1), we found that compared with lean women, obese (aOR = 1.83; 95 % CI: 1.03–3.24) and overweight women (aOR = 1.20; 95 %CI: 0.78–1.83) were more likely to report snoring during pregnancy.

## Discussion

Overall, we found that pregnant women who are overweight and obese have increased odds of sleep disorders. Compared with lean women (<25 kg/m^2^), overweight women (25–29.9 kg/m^2^) had 3.69-fold higher odds of high risk for OSA (95 % CI 1.82–7.50). The corresponding OR for obese women (≥30 kg/m^2^) was 13.23 (95 % CI: 6.25–28.01). Obese women, as compared with their lean counterparts had a 1.61-fold higher odds of poor sleep quality (95 % CI: 1.00–2.63).

In the present study, the prevalence estimates of high risk for OSA were 6.5 % (assessed using Berlin questionnaire). The prevalence of high risk for OSA found in our study is lower than estimates from other studies. In their study among predominantly Hispanic pregnant women in Houston, Texas Antony et al. found a 15.5 % prevalence of high risk for OSA assessed using Berlin questionnaire [4]. The investigators further noted that obesity was associated with a 9-fold increased odds (95 % CI 4.68–17.39) of high risk for OSA compared with normal weight women in that population. In a study of 276 pregnant women in Korea, Ko et al. [20] found a high prevalence of OSA in obese women (43.6 %) compared with non-obese women (32.6 %) (*p* = 0.001). Their findings indicating increased odds of high risk for OSA among overweight and obese pregnant women are in general agreement with our study findings. Other studies conducted among men and non-pregnant women [34–36] report findings that are in agreement with those reported by our team and others [4, 20]. For example, using data from the 2007 Sleep in America Poll of the National Sleep Foundation, Kapsimalis and Kryger [37] noted that the prevalence of high risk for OSA (determined using Berlin questionnaire) was 8.5 % among women with normal BMI while the prevalence estimates were markedly higher among overweight (21 %) and obese (62 %) women. In a recently published study of college students in Chile, Wosu et al. [15] reported, that obese students were 8.26 times-as likely to experience high risk for OSA compared with normal weight students (95 % CI:4.59–14.86) after adjusting for confounders. Another finding that merits consideration is our results that showed obese pregnant women were 1.83-time as likely to report snoring (aOR = 1.83; 95 % CI: 1.03–3.24) during pregnancy as compared with lean women. However, this association did not reach statistical significance for overweight women (aOR = 1.20; 95 % CI: 0.78–1.83). Investigators have documented similar findings and have speculated that pregnant women are more likely to have higher BMI while carrying a lesser proportion of body fat and therefore more likely to meet the criteria of obesity used by the Berlin questionnaire [23]. Taken together, the current data indicate that the Berlin questionnaire might have limited utility in OSA screening due to the inclusion of obesity as one of the classification criteria.

In the last 50 years, the average self-reported sleep duration in the United States has decreased by 1.5 to 2 h while the prevalence estimates of obesity and diabetes have increased with Hispanics and Blacks showing marked increase [38, 39]. A large literature primarily focused on men and non-pregnant women has shown that obesity is related to sleep insufficiency and poor sleep quality [40]. For instance, Chaput et al. (2007) in Canada found that women with short sleep duration (5 to 6 h) were 1.69-times as likely (OR = 1.69; 95 % CI: 1.15–2.39) to be overweight or obese compared with normal sleepers (7 to 8 h) [41]. In Taiwan Hung et al. (2012) noted that being overweight or obese was statistically significantly associated with increased global PSQI scores (*p* < 0.001) [40]. Logue et al. (2014) in an urban family medicine center in the US reported a statistically significant association of poor sleep quality and obesity (*p* = 0.005) independent of age, gender, and ethnicity [42]. It is well established that sleep is altered during pregnancy [43]. Of note, in the second and third trimesters, pregnant women are more likely to have frequent awakenings due to fetal movements, discomfort, backaches as well as frequent urge to urinate due to an enlarged uterus [44] contributing to sleep insufficiency and fragmentation. Poor material sleep quality and other sleep disorders influence not only the mother but also their offspring to adverse cardio metabolic pathology later in life.

In the current study, we found no evidence of an association between BMI and excessive daytime sleepiness after adjustment for possible confounders. Dixon et al. (2007), in their study among 1055 Australian patients presenting for obesity surgery, also found no statistically significant association between ESS scores and BMI [45]. We do not have an explanation for these null findings although it is important to note that the ESS is a global summary score of questions on the risk of daytime sleepiness during different situations during the daytime. The ESS was originally developed to measure one construct—excessive daytime sleepiness [27, 31]. However, an emerging literature has shown that the eight items of ESS do not necessarily assess a unidimensional construct rather two or three different aspects of daytime sleepiness [46]. Hence, the summary score of the all eight items may not be the best index of excessive day time sleepiness. Future studies should look at how individual items might be influenced by obesity/overweight status.

Several plausible and compelling biological mechanisms have been proposed to explain the observed obesity/overweight-sleep disorder associations. Obesity can contribute to many physiological changes that increase the risk of OSA. For example, excessive soft tissue due to obesity, may narrow the pharyngeal airway and reduce lung volume [2]. Additionally, pregnancy induces many physiological changes. These includes enlargement of the uterus which can elevate the diaphragm and alter respiration. These alterations, for instance, may increase the tendency for collapsing the upper airway during sleep [19]. Changes in hormones also predispose pregnant women to episodes of sleep apnea. Notably, increased estrogen concentration has been linked to mucosal edema, and progesterone has been linked to increase respiratory center sensitivity to CO_2,_ therefore causing instability of the respiratory control mechanism [47]. Collectively, these physiological and hormonal changes may interact to increase the obesity-sleep disorders associations.

Our study has several notable strengths including a relatively large sample size, the use of cross-culturally accepted instruments to characterize sleep disorders and rigorously trained research staff administering the questionnaires. However, several important limitations must be considered when interpreting our findings. First, since this was a cross-sectional study, we cannot delineate the temporal relationship between maternal elevated BMI and sleep disorders. We also cannot determine whether sleep disturbances may be attributed to pregnancy related physiological alterations. Second, our findings are based on a population of pregnant women seeking care at a specialized maternal hospital; hence, caution is needed before generalizing the results to other populations. Third, although we used multiple validated questionnaires, these questionnaires may have contributed to some errors in the classification of participants’ sleep disturbances [20]. Use of screening questionnaires for assessing sleep disorders in pregnancy is an important component of sleep health research. The success of screening however, is largely dependent on the accuracy of the questionnaires and use diagnostic procedures, used as the gold standard, to evaluate the effectiveness of screening questionnaires. The gold standard for documenting many sleep disorders including OSA is in-laboratory polysomnography (PSG) assessment. Unfortunately due to cost, complexity and participant burden, use of PSG testing is limited in large-scale epidemiologic studies. Thus use of screening questionnaires with low specificity remains the main modality for ascertaining OSA. Future studies are warranted to develop, refine and enhance the psychometric properties of screening questionnaires and improve their utility for early identification of sleep disorders in pregnancy. The fact that our findings are similar to those that used more invasive, though objective measures of sleep traits (e.g., polysomnography) [48] serve to attenuate some concerns.

Available literature suggests that maternal OSA may be associated with an increased risk of adverse perinatal outcomes [47, 49]. For instance in a meta-analysis of 9795 participants Luque-Fernandez et al. [49] found women with sleep disordered breathing had a 3-fold increased risk of gestational diabetes (OR = 3.06; 95 % CI: 1.89–4.96). Chen et al. [50] found that mothers with OSA were more likely to have low birth weight, preterm birth, and small for gestational age newborns, cesarean section, preeclampsia, gestational diabetes, and gestational hypertension as compared with unaffected mothers. Louis et al. [51] reported that OSA was associated with an increased risk of preterm delivery and maternal morbidity. Additionally, Kamysheva et al. have reported increased odds of antepartum and postpartum depression among women with symptoms of poor sleep [52].

## Conclusion

In conclusion, we observed increased risks of sleep disorders amongst overweight and obese pregnant Peruvian women. These observations, when coupled with earlier report, have important clinical and public health implications because pregnant women with symptoms of OSA are at higher risk of adverse pregnancy and perinatal outcomes [47]. Collectively, our findings and those of others [14] underscore the clinical and public health implications for OSA screening and treatment among reproductive age and pregnant women in low and middle income countries, particularly those undergoing rapid epidemiologic transitions characterized by increased prevalence of excessive adult weight gain.
